# SEOM clinical guideline in ovarian cancer (2020)

**DOI:** 10.1007/s12094-020-02545-x

**Published:** 2021-01-30

**Authors:** A. Redondo, E. Guerra, L. Manso, C. Martin-Lorente, J. Martinez-Garcia, J. A. Perez-Fidalgo, M. Q. Varela, M. J. Rubio, M. P. Barretina-Ginesta, A. Gonzalez-Martin

**Affiliations:** 1 Hospital Universitario La Paz-IdiPAZ, Universidad Autónoma de Madrid (UAM), Paseo de la Castellana 261, 28046 Madrid, Spain; 2 Hospital Universitario Ramón y Cajal, Madrid, Spain; 3 Hospital Universitario 12 de Octubre-i+12, Madrid, Spain; 4 Hospital de la Santa Creu i Sant Pau, Barcelona, Spain; 5 Hospital Clínico Universitario Virgen de la Arrixaca, Murcia, Spain; 6 Hospital Clínico Universitario, Biomedical Research Institute INCLIVA, University of Valencia, Valencia, Spain; 7 Complexo Hospitalario Universitario de A Coruña, A Coruña, Spain; 8 Hospital Universitario Reina Sofía, Universidad de Córdoba (UCO), Córdoba, Spain; 9 Girona Biomedical Research Institute (IdIBGi) and Department of Medical Sciences, Instituto Catalán de Oncología (ICO), Medical School University of Girona, Girona, Spain; 10 Clínica Universidad de Navarra, Madrid, Spain

**Keywords:** Ovarian cancer, Guideline, Diagnosis, Treatment

## Abstract

Despite remarkable advances in the knowledge of molecular biology and treatment, ovarian cancer remains the leading cause of death from gynecologic cancer. In the last decade, there have been important advances both in systemic and surgical treatment. However, there is no doubt that the incorporation of PARP inhibitors as maintenance after the response to platinum-based chemotherapy, first in recurrent disease and recently also in first line, will change the natural history of the disease.

The objective of this guide is to summarize the current evidence for the diagnosis, treatment, and follow-up of ovarian cancer, and to provide evidence-based recommendations for clinical practice.

## Introduction

Epithelial ovarian cancer (OC) remains the 5th cause of death in women and the first cause of death due to gynecological cancer. In the last two decades, we have assisted to achievements in the knowledge of molecular biology, surgical outcome, chemotherapy administration, and implementation of antiangiogenic therapy that have translated into clinically significant improvements in the time of disease control and overall survival in some cases. More recently, the incorporation of Poly ADP Ribose Polymerase (PARP) inhibitors as maintenance after response to platinum-based chemotherapy seems to start changing the natural history of the disease and the aim of decreasing the mortality is becoming a reality. This SEOM guideline is providing updated evidence-based recommendation for the current treatment of ovarian, primary peritoneal and fallopian tube cancer, globally considered as OC along the guideline.

## Methodology

This guideline has been developed with the consensus of ten oncologists, with high expertise in OC treatment, from the cooperative group GEICO (Spanish Group for Investigation in Ovarian Cancer) and SEOM (Spanish Society of Medical Oncology). To assign a level and quality of evidence and a grade of recommendation to the different statements of this treatment guideline, the Infectious Diseases Society of America-US Public Health Service Grading System for Ranking Recommendations in Clinical Guidelines was used.

## Diagnosis and staging

Early-stage OC is frequently associated to non-specific symptoms, but in late-stages ascites, abdominal disorders and/or pleural effusion are common. In women with symptoms suggestive of OC, physical examination, laboratory testing with CA 125 and pelvic ultrasonography are recommended at a first level [II, A]. High levels of HE4 identify malignancy with a similar sensitivity than CA 125, but with higher specificity. Both CA 125 and HE4 are included in the Risk Ovarian Malignancy Algorithm (ROMA), used for calculating the risk of malignancy of adnexal masses.

In patients with presumed OC, computed tomography (CT) imaging of the thorax, abdomen and pelvis will define the extent of the disease and provide information to plan treatment options [II, A]. Magnetic resonance imaging (MRI) and Positron Emission Tomography (PET)-CT are not included in the routine preoperative staging but could improve the accuracy of the evaluation of the advanced disease [II, B]. Laparoscopic surgery is a mainstay not only in staging of OC but also in pathological diagnosis [II, B]. FIGO 2014 is the staging system currently recommended [1] (Table 1).

**Table 1:** 2014 FIGO staging system for ovarian, fallopian tube, and peritoneal cancer^1^

Stage I	Tumour confined to ovaries or fallopian tube(s)
IA	Tumour limited to one ovary (capsule intact) or fallopian tube; no tumour on ovarian or fallopian tube surface; no malignant cells in the ascites or peritoneal washings
IB	Tumour limited to both ovaries (capsules intact) or fallopian tubes; no tumour on ovarian or fallopian tube surface; no malignant cells in the ascites or peritoneal washings
IC1	Tumour limited to one or both ovaries or fallopian tubes, with surgical spill
IC2	Tumour limited to one or both ovaries or fallopian tubes, with capsule ruptured before surgery or tumour on ovarian or fallopian tube surface
IC3	Tumour limited to one or both ovaries or fallopian tubes, with malignant cells in the ascites or peritoneal washings
Stage II	Tumour involves one or both ovaries or fallopian tubes with pelvic extension or primary peritoneal cancer
IIA	Extension and/or implants on uterus and/or fallopian tubes and/or ovaries
IIB	Extension to other pelvic intraperitoneal tissues
Stage III	Tumour involves one or both ovaries or fallopian tubes, or primary peritoneal cancer, with cytologically or histologically confirmed spread to the peritoneum outside the pelvis and/or metastasis to the retroperitoneal lymph nodes
IIIA1	Positive retroperitoneal lymph nodes only (cytologically or histologically proven)
IIIA1(i)	Metastasis up to 10 mm
IIIA1(ii)	Metastasis more than 10 mm
IIIA2	Microscopic extrapelvic (above the pelvic brim) peritoneal involvement with or without positive retroperitoneal lymph nodes
IIIB	Macroscopic peritoneal metastasis beyond the pelvis up to 2 cm in greatest dimension, with or without metastasis to the retroperitoneal lymph nodes
IIIC	Macroscopic peritoneal metastasis beyond the pelvis more than 2 cm in greatest dimension, with or without metastasis to the retroperitoneal lymph nodes (includes extension of tumour to capsule of liver and spleen without parenchymal involvement of either organ)
Stage IV	Distant metastasis excluding peritoneal metastases
IVA	Pleural effusion with positive cytology
IVB	Parenchymal metastases and metastases to extra-abdominal organs (including inguinal lymph nodes and lymph nodes outside of the abdominal cavity)

## Pathology and biomarkers

According to the WHO classification, there are five major subtypes of epithelial ovarian carcinoma: high-grade serous (HGSC), low-grade serous (LGSC), clear cell (CCC), endometrioid (EC), and mucinous (MC). The use of an immunohistochemical (IHC) panel including Wilms Tumor 1 (WT1), p53, progesterone receptor (PR), and Napsin A (NAPSA) has been suggested to assist in the diagnosis of cases in which the histological type is difficult to establish [2] [II, A]. The OC subtypes differ not only in IHC expression but also in molecular features (Table 2). Furthermore, in non-mucinous tumors, a study of germinal (and somatic if possible) *BRCA* mutation must be completed after pathological diagnosis due to its implications on hereditary counseling, prognostic information, and therapeutic strategy [I, A]. For therapeutic decisions, it would be desirable to have also information on tumor homologous recombination (HR) status [I, A].

**Table 2:** Immunohistochemical panel for diagnosis of the subtypes of ovarian cancer

	Pax8	WT1	P53	ER	NAPSA	Ki67
HGSC	+	+	+ (diffuse)	+	−/ +	High
LGSC	+	+	–	+	–	Low
CCC	+	–	−/ +	–	+	N/A
EC	+	–	–	+	−/ +	N/A
MC	−/ +	–	–	−/ + (focal)	–	N/A

*HGSC* high-grade serous carcinoma, *LGSC* low-grade serous carcinoma, *CCC* clear cell carcinoma, *EC* endometrioid carcinoma, *MC* mucinous carcinoma, *WT1* Wilms Tumour 1, *ER* estrogen receptor, *NAPSA* naspin A, *N/A* not applicable

## Early stages

### Surgery

The treatment for early-stage OC (stages I-II) consists of a staging surgery that includes hysterectomy with bilateral salpingo-oophorectomy, bilateral pelvic and para-aortic lymphadenectomy, omentectomy, random peritoneal biopsies, peritoneal washing, and careful inspection and palpation of all peritoneal surfaces, the serosa of the entire digestive tract and the bowel mesentery [II, A]. Lymph node dissection can safely be omitted in grade I mucinous tumors. Both open and laparoscopic approaches are acceptable as long as an experienced gynaecologic oncologist performs all the procedure [3] [II, A]. In patients referred to after incomplete surgery, a re-staging procedure should be offered [III, A].

Fertility-sparing surgery, based on a unilateral salpingo-oophorectomy and complete surgical staging can be safely offered to all stage IA, and IC1 low-grade ovarian carcinomas [II, A].

### Adjuvant treatment

Adjuvant platinum-based chemotherapy after staging surgery is indicated in high-risk early stages (IA/B high grade, IC-IIA) [I, A]. The benefit is uncertain and should be discussed on individually in: Grade 1–2 EC stage IB/IC, MC with expansile invasion stage IB/IC, MC with infiltrative invasion stage IA, LGSC stage IB/IC, and CCC stage IA-IC1 [4].

The recommended regimen is paclitaxel-carboplatin, but single-agent carboplatin is also considered a reasonable option [I, A]. 6-cycle regimen is recommended in HGSC, but the optimal duration remains controversial in the rest of histologic subtypes, in which three cycles can be accepted [4] [I, A].

## Advanced disease: front-line treatment

### Cytoreductive surgery

Cytoreductive surgery plays a crucial role in the treatment of advanced OC (stages III and IV). The presence of visible residual disease after cytoreduction is a major prognostic factor with an important negative impact on survival. Thus, the goal must be to obtain a complete cytoreduction [II, A]. In this context surgical effort must be maximal and may include histerectomy, double anexectomy, omentectomy, extensive peritonectomy, bowel resection or excision of any enlarge nodes. Due to the recent results of Lion trial lymphadenectomy in primary completely debulked advanced OC with clinically negative lymph nodes is not further recommended [5] [I, A].

Several studies and meta-analysis suggest that the expertise of the surgeon is of utmost importance in the outcomes and thus the recommendation is that patients should be operated in highly experienced centers [6] [II, A].

### Neoadjuvant chemotherapy

The EORTC55971 trial and the CHORUS trial showed a similar progression-free survival (PFS) and overall survival (OS) for patients with stage IIIC or IV disease receiving neoadjuvant chemotherapy (NACT) and interval debulking surgery (IDS) compared with primary debulking surgery (PDS). In spite of these non-inferiority results, these aforementioned trials have been criticized because of the median OS, mean operative time and low rates of optimal cytoreduction.

Therefore, both approaches (PDS or NACT followed by IDS) can be considered valid, although PDS remains as the preferred primary treatment when complete cytoreduction is feasible and patient is operable [4] [I, A].

### Chemotherapy regimen and route of administration

Standard post-operative treatment in advanced OC consists of a combination of carboplatin (AUC 5–6) and paclitaxel (175 mg/m^2^) every 3 weeks for six cycles [I, A].

Incorporation of weekly chemotherapy into first-line treatment does not improve PFS or OS in the population of western countries [7]. Moreover, single agent carboplatin or weekly chemotherapy could have even worse outcomes in vulnerable elderly patients, as shown by EWOC-1 trial, so that 3-weekly regimen remains the standard for all OC populations [I, A].

Three large randomized studies (GOG 104, GOG 114, and GOG 172) and one meta-analysis have found clinically significant improvements in PFS and OS with intraperitoneal (IP) chemotherapy [8]. However, in the GOG 252 trial the duration of PFS was not significantly increased with either IP regimen when bevacizumab was incorporated in all arms, and IP cisplatin arm was associated to higher toxicity [9]. Therefore, and according to last ESMO-ESGO consensus, currently IP chemotherapy is not a standard of care [4] [III, A]. Nevertheless, it could still be considered in selected patients (stage III, < 1 cm residual disease) as long as bevacizumab is not used [I, B].

A randomized phase III trial evaluating hyperthermic intraperitoneal chemotherapy (HIPEC) after IDS showed a better OS for HIPEC arm. However, this trial has received significant methodological criticisms, so HIPEC cannot be considered a standard treatment and should not be offered out of clinical trials [4] [I, C].

### Bevacizumab

Two large randomized studies (GOG 218 and ICON 7) have reported that bevacizumab added to the initial chemotherapy followed by a maintenance period improves PFS in comparison with standard chemotherapy alone [10, 11]. None of these trials showed an OS benefit in the overall study populations but post hoc subgroup analysis indicated statistically significant OS benefit in patients with stage IV disease in GOG 218 (18) and patients at high risk of progression (defined as FIGO stage III with > 1 cm residual disease after PDS or stage IV) in the ICON7 trial.

Bevacizumab (15 mg/kg or 7.5 mg/kg every 3 weeks for a maximum of 15 months) should be considered in addition to carboplatin and paclitaxel, and it is especially recommended in patients with stage III and residual disease or stage IV [I, A].

### Maintenance treatment with PARP inhibitors

Four randomized phase III trials (SOLO-1, PRIMA, PAOLA-1 and VELIA) have shown that maintenance treatment with PARP inhibitors (PARPi) after response to front-line platinum-containing regimens increased significantly the median PFS in HGSOC [12–15]. Table 3 summarizes differences in the design between these trials and the results in the ITT and the different biomarker subgroup populations. All trials have shown a remarkable and unprecedented benefit in BRCAmut. In addition, PAOLA-1 and PRIMA demonstrated also a significant benefit in HR deficient (HRD) population. Finally, only PRIMA showed a benefit in the HR proficient (HRP) subgroup although of lesser magnitude. The benefit observed with PARPi is sustained along the follow-up as demonstrated by the impact on PFS2, as well as by the results after a 5-year follow-up of the SOLO-1 showing that almost 50% of patients remain progression-free in contrast to 21% in the control arm.

**Table 3:** Characteristics of patients and outcomes of phase III clinical trials with PARP inhibitors in first line

	SOLO-1	PAOLA-1	PRIMA	VELIA
Intervention arm	Olaparib 300 mg BID	Olaparib 300 mg BID + Bevacizumab	Niraparib 300 or 200 mg QD	Veliparib 150 BID C 1–6 and 400 mg BID cycle 7–36
Control arm	Placebo	Bevacizumab	Placebo	Placebo
Duration PARPi	2 years	2 years	3 years	36 cycles (27 months)
FIGO				
Stage III	85–80%	69–70%	62–65%	77–78%
Stage IV	15–20%	30–31%	34–38%	22–23%
NACT	NR	41–42%	63–67%	26–29%
Residual disease	21–22%	29–41%	100% in stage III with PDS	30–32%
Biomarkers	100% *BRCA*m	29% *BRCA*m 48% HRD	31% *BRCA*m 51% HRD	27–31%*BRCA*m 63% HRD
HR in overall population	NA	0.59	0.62	0.68
HR in *BRCA*m	0.30	0.31	0.40	0.44
HR in HRD and *BRCA*wt	NA	0.43	0.50	*p* = n.s.
HR in HRP	NA	*p* = n.s.	0.68	*p* = n.s.

*NACT* neoadjuvant chemotherapy, *HR* hazard ratio, *BRCA*m *BRCA* mutated; *BRCA*wt *BRCA* wild type, *HRD* homologous recombination deficiency, *HRP* homologous recombination proficiency, *PDS* primary debulking surgery, *NA* not Applicable, *n.s.* not significant

Based on these results, olaparib (with or without bevacizumab) or niraparib after partial or complete response to first-line platinum-based chemotherapy are highly effective in *BRCA*-mutated patients and strongly recommended [I, A].

According to PAOLA-1 and PRIMA results, niraparib or olaparib-bevacizumab are also highly recommended for patients with HRD tumors [I, A]. In the HRP subgroup maintenance with niraparib can also be considered although bevacizumab remains as a reasonable alternative [I, B].

## Recurrent disease

Approximately, 80–85% of patients with advanced OC will relapse in the first 10 years after the diagnosis [16]. When planning treatment for recurrent disease, first considerations must be willingness of the patient to receive further therapy and performance status (PS). Next step is to decide if platinum might be the best option taking into account the following factors. Conversely, the traditional and arbitrary classification as platinum-sensitive and platinum-resistance has been abandoned during the Fifth Ovarian Cancer Consensus Conference (OCCC) of the GCIG and the ESMO-ESGO consensus [4, 17].

### Factors to consider when selecting therapy

Depending on the tumour: Site and extension of the disease, histological subtype, *BRCA* mutation status.

Depending on the patient: Treatment-free interval. Platinum-free interval (TFIp) has classically been considered a predictive factor of response to platinum-rechallenge. Also TFInp (non-platinum) or TFIb (biological) should be considered as well as number of previous therapies, residual toxicity, patient preferences and comorbidities with special attention to geriatric population [17].

### Surgery for relapsed ovarian cancer

The randomized Desktop III trial has shown a significant OS benefit of surgery at relapse among patients accomplishing AGO score. Therefore, surgery should be recommended for patients with TFIp > 6 months, no residual disease after first surgery, good PS (0–1), and absence or less than 500 ml of ascites [18] [I, A]. In addition, PET-CT could improve the selection of candidates for secondary cytoreduction [19] [II, B].

### Systemic treatment when platinum might be the best option

A platinum-based combination (with paclitaxel, gemcitabine or pegylated liposomal doxorubicin -PLD-) is associated with a longer PFS and OS compared to single-agent platinum. None of these combinations can be considered superior in terms of efficacy; the doublet selection should be based on the toxicity profile.

A randomized phase III trial of bevacizumab combined with carboplatin-gemcitabine, in patients in first relapse who have not been treated with antiangiogenic therapy, has shown a benefit in response rate (RR) and PFS [20]. The combination of bevacizumab with carboplatin and paclitaxel in this setting has also shown improvement in PFS [21]. Recently, the combination carboplatin-PLD and bevacizumab have shown benefit in PFS and OS over carboplatin-gemcitabine and bevacizumab [22].

Three PARP inhibitors (olaparib [23] niraparib [24] and rucaparib [25]) have shown benefit in PFS as maintenance treatment after response to platinum based therapy in relapsed ovarian cancer. The magnitude of benefit is greater, but not limited, to patients with *BRCA* mutation. For *BRCA*-mutated patients maintenance treatment with olaparib improves PFS (HR 0.30) and OS (HR 0.74) with an improvement of 12.9 months in median OS vs placebo. Niraparib and rucaparib have also shown positive results in phase III trials, not only in *BRCA*-mutated patients but also in *BRCA* wild type (wt), regardless the status of HR.

Therefore, when platinum might be the best option both platinum-based combination with bevacizumab and platinum-based combination followed by PARPi are optimal options [I, A]. Based on the higher RR with the addition of bevacizumab, this option would be indicated for symptomatic patients, without *BRCA* mutation, who did not receive bevacizumab at first line. For the rest of patients, platinum-based combination followed by PARPi would be the preferred option [III, A]. For *BRCA*-mutated patients olaparib, niraparib or rucaparib can be used, and for *BRCA*wt patients niraparib or rucaparib are the available options [I, A].

If *BRCA* is mutated, monotherapy with rucaparib (not as maintenance) is also an alternative for patients with no previous PARPi treatment, who have been treated with two or more prior lines of platinum-based chemotherapy, and who are unable to tolerate further platinum-based chemotherapy [II, B].

In patients with TFIp > 6 months who cannot receive platinum-based therapy, or after previous use of PARPi and at least two platinum regimens, the combination trabectedin plus PLD could be an option [I, B].

### Systemic treatment when platinum might not be the best option

Patients progressing on platinum-based therapy or after a short treatment-free interval of platinum are not considered eligible for re-challenge with platinum. This is an unmet medical need and when possible, patients should be included in clinical trials. Cytotoxic agents, such as weekly paclitaxel, PLD, gemcitabine and topotecan, have shown modest activity in phase III randomized trials, with an average RR of 10–15% and median OS in the range of 9–12 months. Accordingly, sequential cytotoxic single-agent therapy is the best palliative option and quality of life is the most important endpoint**.** Nevertheless, patients with poor PS could be considered only for best supportive care.

For patients who have not received prior bevacizumab, the addition of the latter to weekly paclitaxel, PLD, or topotecan has shown to improve PFS. The combination weekly paclitaxel and bevacizumab was especially active in Aurelia trial, being the preferred option when possible [26].

In summary, when platinum might not be not the best option single-drug therapy, or in combination with bevacizumab if not previously received, is recommended [I, A].

## Follow-up

Table 4 summarized our recommendations for follow-up. Although performing a routine imaging is controversial we recommended to do it at least every 6 months. It is important to review any new symptom reported by the patient and to perform a physical examination in each visit.

**Table 4:** Ovarian cancer follow-up recommendations

	0–2 years from end of treatment	2–5 years from end of treatment	5–10 years from end of treatment
Review of symptoms	Every 3 months*	Every 6 months	Yearly
Physical examination	Every 3 months*	Every 6 months	Yearly
Blood test + CA 125	Every 3 months*	Every 6 months	Yearly
CT scan	Every 3–6 months	Every 6 months	Yearly

*In early-stage ovarian cancer these procedures could be performed every 6 months from the beginning
